# Late Autism Spectrum Disorder (ASD) Diagnosis: A New Perspective on Life

**DOI:** 10.1192/j.eurpsy.2025.1644

**Published:** 2025-08-26

**Authors:** K. Tolvaišaitė, K. Butkutė-Šliuožienė, A. Kizienė

**Affiliations:** 1 Psychiatry, Lithuanian University of Health Sciences; 2 Psychiatry, Kaunas Hospital of the Lithuanian University of Health Sciences, Kaunas, Lithuania

## Abstract

**Introduction:**

Autism Spectrum Disorder is usually diagnosed in early childhood, but an increasing number of adults are identified as autistic later in life (Huang et al., 2020). Many experience frustration due to missed early intervention opportunities. The lack of recognition of their condition during childhood or adolescence may have contributed to secondary mental health issues, such as anxiety, depression or low self-esteem, complicating the adjustment process after diagnosis (Bargiela et al., 2019). However, providing appropriate support can enhance their quality of life and promote better mental health outcomes.

**Objectives:**

To assess the patient’s clinical case to identify autism spectrum disorder in older adulthood.

**Methods:**

A 23-year-old male patient complained of an inability to concentrate, stress and fear when around people, and difficulties in social situations. Objectively observed: stereotyped movements, noticeable anxiety, avoidance of eye contact, non-compliance with social rules, specific language use and slow thinking. The patient had no history of diagnosed psychiatric illness. Since childhood, he has disliked the feeling of clothes touching his body, physical contact with others, making eye contact. The patient also exhibited impaired social development, being unable to initiate and maintain relationships with peers. Stereotypical, repetitive movements, sensory processing deviations have been observed since childhood. The patient has various phobias from a young age. Psychological examination revealed insufficient attention with observed fluctuations, impaired attention-shifting ability. The pace is very slow, the thinking is characterized by an average level of generalization, concreteness, and stereotypy. Personality traits included rigidity, depression, compulsiveness, internal tension. Based on the clinical picture and psychological tests, the patient was diagnosed with F84.0 Autistic Disorder, according to the International Classification of Diseases, 10 th Revision. In this case, the following methods were used in the assessment: Kraepelin’s and Schulte’s methods, pictograms, the 4-1 method, the Childhood Autism Rating Scale.

**Results:**

The literature highlights that ASD involves difficulties in social interaction, repetitive behaviors, sensory sensitivities, and distinct thinking patterns. Up to 90% of individuals with ASD experience sensory irregularities, which is consistent with this case, as the patient shows social challenges, repetitive movements, slow thinking, and sensory issues. Diagnosing autism in adulthood can improve quality of life by fostering understanding and access to support. In this case, the patient’s past social and workplace difficulties may be attributed to autism, guiding the development of effective support strategies.

**Conclusions:**

In conclusion, while a late autism diagnosis can be life-changing, it also comes with unique challenges that must be addressed through appropriate support.

**Disclosure of Interest:**

None Declared

